# Effectiveness of interventions to reduce household air pollution from solid biomass fuels and improve maternal and child health outcomes in low- and middle-income countries: a systematic review protocol

**DOI:** 10.1186/s13643-021-01590-z

**Published:** 2021-01-20

**Authors:** Katherine E. Woolley, Emma Dickinson-Craig, Suzanne E. Bartington, Tosin Oludotun, Bruce Kirenga, Shelton T. Mariga, Telesphore Kabera, April Coombe, Francis D. Pope, Ajit Singh, William R. Avis, Rosie Day, David Warburton, Semira Manaseki-Holland, David J. Moore, G. Neil Thomas

**Affiliations:** 1 Institute of Applied Heath Research, University of Birmingham, Birmingham, UK; 2 Makerere University Lung Institute, College of Health Sciences, Mulago Hospital, Kampala, Uganda; 3 University of Rwanda College of Science and Technology, Kigali, Rwanda; 4 School of Geography, Earth and Environmental Sciences, University of Birmingham, Edgbaston, Birmingham, UK; 5 International Development Department, University of Birmingham, Edgbaston, Birmingham, UK; 6 Children’s Hospital Los Angeles, University of Southern California, Los Angeles, USA

**Keywords:** Indoor air pollution, Interventions, Low- and middle-income countries, Pregnancy outcomes, Child health, Maternal health, Environmental health, Biomass, Infant health

## Abstract

**Background:**

A variety of public health interventions have been undertaken in low- and middle-income countries (LMICs) to prevent morbidity and mortality associated with household air pollution (HAP) due to cooking, heating and lighting with solid biomass fuels. Pregnant women and children under five are particularly vulnerable to the effects of HAP, due to biological susceptibility and typically higher exposure levels. However, the relative health benefits of interventions to reduce HAP exposure among these groups remain unclear. This systematic review aims to assess, among pregnant women, infants and children (under 5 years) in LMIC settings, the effectiveness of interventions which aim to reduce household air pollutant emissions due to household solid biomass fuel combustion, compared to usual cooking practices, in terms of health outcomes associated with HAP exposure.

**Methods:**

This protocol follows standard systematic review processes and abides by the PRISMA-P reporting guidelines. Searches will be undertaken in MEDLINE, EMBASE, CENTRAL, WHO International Clinical Trials Registry Platform (ICTRP), The Global Index Medicus (GIM), ClinicalTrials.gov and Greenfile, combining terms for pregnant women and children with interventions or policy approaches to reduce HAP from biomass fuels or HAP terms and LMIC countries. Included studies will be those reporting (i) pregnant women and children under 5 years; (ii) fuel transition, structural, educational or policy interventions; and (iii) health events associated with HAP exposure which occur among pregnant women or among children within the perinatal period, infancy and up to 5 years of age. A narrative synthesis will be undertaken for each population-intervention-outcome triad stratified by study design. Clinical and methodological homogeneity within each triad will be used to determine the feasibility for undertaking meta-analyses to give a summary estimate of the effect for each outcome.

**Discussion:**

This systematic review will identify the effectiveness of existing HAP intervention measures in LMIC contexts, with discussion on the context of implementation and adoption, and summarise current literature of relevance to maternal and child health. This assessment reflects the need for HAP interventions which achieve measurable health benefits, which would need to be supported by policies that are socially and economically acceptable in LMIC settings worldwide.

**Systematic review registration:**

PROSPERO CRD42020164998

## Background

Household cooking, heating and lighting with solid biomass fuel (e.g. wood, dung, charcoal, crop residues) [1] is common in low- and middle-income countries (LMICs) [2] worldwide, producing hazardous levels of household air pollution (HAP) (e.g. carbon monoxide (CO) and particulate mMatter (PM)) [3], and exposure to results in significant morbidity and mortality. The greatest burden of HAP exposure is recognised to be among women of child-bearing age [4] and children under 5 years, due to a disproportionate amount of time spent in the house, with women performing or assisting with household duties [5]. Intrauterine, infancy and early childhood are critical periods of organ development when individuals are particularly vulnerable to the harms of HAP exposure [6]. Adverse health events associated with HAP exposure can occur throughout the life course from conception to old age, but specifically during pregnancy, with evidence for increased risk of low birth weight, preterm birth, stillbirth, gestational hypertension, intrauterine growth retardation (IUGR) and perinatal mortality [7]. Early life health events among infants and children under 5 years include increased risks of acute lower respiratory infection (ALRI), asthma, otitis media, impaired neurodevelopment and all-cause mortality [8, 9]. In context, 31.75 per 100,000 acute respiratory infection (ARI) deaths and 11.68 per 100,00 preterm birth deaths were attributable to HAP globally in 2019 [10].

Ultimately, economic development is associated with clean fuel transitions (e.g. to ethanol, liquid petroleum gas (LPG), electricity), which are fuels that have been recognised to reduce HAP levels to below World Health Organization indoor air quality (WHO-IAQ) guideline levels (CO 7 mg/m^3^ 24-h average, PM_2.5_ 25 mg/m^3^ annual average) [11]; however, socio-cultural factors can contribute to fuel/stove stacking and mixing (where traditional fuels/stoves are used alongside the intervention) which may reduce the efficacy of clean fuel policy implementation [12]. Interim interventions, prior to sustained cleaner fuel availability, to mitigate HAP exposure levels within the household setting are broad ranging, including improved cookstoves (ICS) (e.g. rocket stoves, plancha) [13], solar stoves [14], improved biomass fuels (e.g. briquettes, biomass pellets) [13] and behavioural changes (e.g. removal of the child from the cooking area, outdoor cooking, opening windows) [12]. LPG, for example, has the potential to reduce HAP levels below the WHO-IAQ guideline levels; however, not all interventions achieve this [15–17] or interim targets (PM_2.5_ 35 mg/m^3^ annual average) [18] and are therefore typically harm mitigation measures, with some interventions not reducing exposure at all. In addition, there are often multiple barriers [19] to implementation, uptake and sustained use of interventions, such as fuel affordability and accessibility, cultural and social preferences or lack of relevant infrastructure [20]. Previous systematic reviews have detailed the change in HAP levels [13] and health outcomes (low birth weight, preterm birth, perinatal mortality, paediatric ALRI and chronic obstructive pulmonary disease (COPD)) attributed to ICS interventions [21], in addition to systematic reviews investigating a wider range of HAP interventions for specified symptoms (e.g. blood pressure) [22] and general respiratory and non-respiratory health outcomes [17]. However, to the best of our knowledge, there is a paucity of evidence synthesis concerning the overall benefits to maternal and child health outcomes arising as a consequence of household solid biomass fuel interventions.

The objective of the systematic review protocol outlined here is to assess, among pregnant women, infants and children (under 5 years) in LMIC settings, the effectiveness of interventions which aim to reduce household air pollutant emissions due to household solid biomass fuel combustion, compared to usual cooking practices, in terms of health outcomes associated with HAP exposure. In addition, any information regarding measures of sustained uptake of the intervention within the selected studies will be extracted and discussed. The findings will inform future intervention studies and policy changes, by generating knowledge of effectiveness for achieving improved pregnancy, perinatal, infant and under 5 years child health outcomes in resource-poor settings worldwide.

## Methods

Established systematic review methods will be used. This protocol has been registered on the International Prospective Register of Systematic Reviews (PROSPERO) (ID: CRD42020164998) [23] and is presented in accordance with Preferred Reporting Items for Systematic Reviews and Meta-Analyses Protocol (PRISMA-P) guidelines [24].

### Eligibility criteria

The following Population-Intervention-Comparator-Outcome-Study design (PICOS) criteria will be used to determining primary study inclusion.

#### Population

Pregnant women (no limitation to trimester or number of previous pregnancies), children in infancy and children under the age of 5 years who are exposed to HAP originating from biomass solid fuel sources, used for cooking, heating and lighting within LMIC settings (World Bank definition 2020) [25]. HAP exposure can be determined through direct objective measurement (e.g. personal, kitchen area) of pollutant concentration (e.g. PM, CO) or use of a proxy measure (e.g. self-reported biomass fuel use, classification of ‘cleaner’ and ‘dirty’ fuels by household survey).

#### Intervention

Any intervention implemented which aims to reduce household air pollution emissions arising from indoor cooking or heating using solid biomass fuel. This includes interventions such as those which seek to improve access and take-up to cleaner fuels (e.g. refined biomass, ethanol, LPG, solar, electricity); structural interventions such as improved cookstoves (ICS), inbuilt stoves (e.g. plancha), ventilation and chimney hood; fuel policy; and behavioural/educational interventions (e.g. moving cooking outside, reducing time spent in the kitchen, removing children from the cooking area during cooking, altering fuel or food preparation). There will be no limitation to the length of duration of interventions or timing of deployment of intervention (e.g. anytime during pregnancy through to the fifth year of a child’s life).

#### Comparator

Alternative HAP intervention (e.g. any other intervention within inclusion criteria) or no intervention (e.g. exposure to standard HAP through using the current method of cooking, heating or lighting).

#### Outcomes

Health outcomes relating to pregnancy and perinatal period (e.g. IUGR, birth weight, preterm birth, pre-eclampsia, pregnancy-induced hypertension, maternal mortality, perinatal/infant mortality, stillbirth and miscarriage) and early life (e.g. upper and lower respiratory tract infections, pneumonia, asthma, respiratory distress syndrome, otitis media, impaired neurodevelopment, mortality and burns) which have been previously associated with HAP exposure. There will be no limits to the follow-up duration of outcome measures.

#### Study type

Eligible study designs are randomised control trials (RCTs), non-randomised control trials and quasi-experimental or natural experimental studies (before-after studies, interrupted time-series studies). Time-series or before-and-after studies will need to compare the same health outcomes in the same population pre- and post-intervention. It is recognised that before-and-after studies assessing pregnancy outcomes are unlikely to exist due to the difficulties in assessing changes in pregnancy outcomes within subsequent pregnancies, but will not be excluded if present.

### Exclusion

Any study that did not meet the inclusion criteria in all five areas (population, intervention, comparator, outcomes and study design) will be excluded.

### Information sources

The following databases will be used to search for published, in progress and grey literature: MEDLINE (in process and 1947–date), EMBASE (1947–present), The Cochrane Central Register of Controlled Trials (CENTRAL), WHO International Clinical Trials Registry Platform (ICTRP) [26], ClinicalTrials.gov, The Global Index Medicus (GIM) [27] and Greenfile [28]. Furthermore, the use of manual searches of all reference lists in the included studies and previous systematic reviews related to the topics will ensure capture of all available literature. The systematic reviews will be identified whilst screening the search results for included studies and additionally searching Epistemonkios [29].

### Search strategy

The search strategy, where the database platform allows, will include free-text terms and index terms that are contained within the following structure: “Population” AND (“Intervention” (“Household Air pollution” AND “LMICs”)) (Appendix), with population being defined as pregnant women and children under 5 and interventions being any intervention that aims to reduce the level of household air pollution. There will be no restrictions in place for the date of publication, language of publication, type of publication (e.g. conference abstracts) or type of study design.

### Study records

#### Selection process

Two reviewers (KEW, EDC) will independently conduct article selection using the eligibility criteria, within Mendeley, after removal of duplicates. Relevant articles will be determined initially by title and abstracts, followed by retrieval and full paper assessment for selection of papers as per the inclusion criteria, with reasons for exclusion noted at each stage (including the screening stage). Authors will be contacted for clarification if required. Any difference in selected articles between reviewers will be discussed using a third independent reviewer (SEB) to adjudicate any remaining disagreements. The selection process will be graphically illustrated using a PRISMA flow diagram [24].

#### Data extraction

Data will be independently extracted from included studies by two reviewers (KEW, EDC) using an adapted (to type of study design) Cochrane Public Health Group data extraction form [30], in a Microsoft Excel spreadsheet (Microsoft Cooperation). The data extraction form will include critically appraisal of paper quality within the assessment process. Extracted data will include, but not be limited to:

- Study characteristics
  - ○ Article title, author and year, geographical setting, study population characteristics (sex, age, residential setting), inclusion and exclusion criteria, funding sources
- Intervention details
  - ○ Type of interventions and comparators, length of intervention, baseline imbalances, issues with uptake and adoption
  - ○ Type of air pollution measurements, length of measurement, equipment used and results (if any)
- Health outcomes
  - ○ Health outcomes and definitions, method of measurement/classification scales, appropriateness of method, time points measured

Given the likely variability between studies included in the review, in terms of design, population, intervention, comparator, outcomes and data type, the data extraction process will be piloted and then modified if required. Any differences between reviewers in data extracted will be discussed and using a third independent reviewer (SEB) to adjudicate any remaining disagreements.

#### Quality and bias assessment

Risk of bias will be assessed using the Effective Public Health Practice Project (EPHPP) quality assessment tool for quantitative studies [31] by two reviewers independently (KEW, EDC), assigning low, medium and high risk of bias for each individual study. For trials where a parallel control group is used, it is accepted that random allocation and the blinding of participants and outcome assessor may not be always possible, due to the nature of the interventions and settings.

#### Data synthesis

**Fig. 1:**
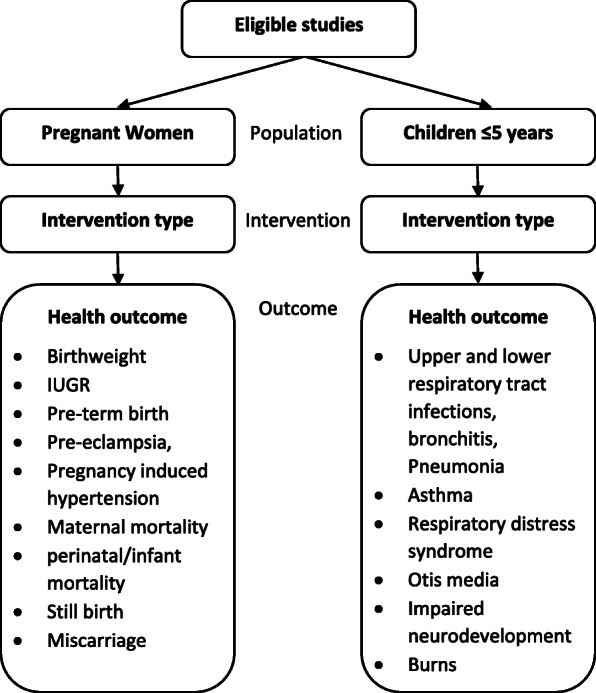
Flow diagram of study grouping (population-intervention-outcome) for synthesis. *IUGR* intrauterine growth retardation

A narrative synthesis will be undertaken for each population-intervention-outcome triad (as indicated in Fig. 1) stratified by study design. Data collected will be tabulated reporting study characteristics, intervention, HAP exposure measurements (if any) and outcome details. It is likely that data may be reported in a mixture of formats for the same outcome (e.g. continuous data mean, proportion meeting a fixed change, risks/relative risks, odds ratios). In addition, there will be a range of health outcomes reported, as well as a mixture of type of interventions, geographical regions and social contexts reported, which are likely to not be directly comparable.

Following on from the narrative analysis, meta-analysis will be considered within each triad, for each outcome measure, stratified by study design and the type of data available for the outcome. Clinical and methodological homogeneity within each triad will be used to determine the feasibility for meta-analysis where two or more studies in the same grouping report data in the same format at the same/similar time points. Any meta-analysis will be undertaken using a random effects model, due to an assumption that the studies represent a distribution of true effects. Determination of the level of between-study variation not attributable to chance will be calculated and displayed as an *I*^*2*^ value with 95% confidence interval.

It is not anticipated that there will be more than a few studies in each meta-analysis, if even such an analysis is possible. The potential for additional sub-group analysis, sensitivity analysis or the assessment for the existence of small study effects using a funnel plot, will likely not exist. Risk of bias information will be used descriptively to contextualise the findings for each outcome whether a meta-analysis is undertaken or not. Recommendation for the improved conduct of studies in the field will be made.

## Discussion

Alternatives to standard practices of household biomass fuel use for cooking, heating and lighting are required within LMICs to combat the morbidity and mortality presented by HAP, with implications for maternal and child health and sustainable economic development. National and local policymakers increasingly recognise the need for effective policy changes to mitigate the health burden associated with HAP exposure; however, there is a lack of evidence regarding affordable, effective and culturally acceptable interventions. This may restrict the progress of such changes, notably in countries which lack widespread access to mains electricity or gas for household cooking, heating and lighting, in addition to limits in transferability of effectiveness of interventions from one context to another. Harm mitigation approaches would bridge the gap before there is widespread affordable access to electricity or gas, but the efficacy of such an intervention requires evaluation. Therefore, this proposed review aims to report the contemporary scientific evidence concerning the effectiveness of HAP mitigation interventions to improve maternal and child health, thus identifying existing research gaps and informing future research and impact activities.

## Data Availability

Not applicable, as no data was generated for this article

## Appendix

### MEDLINE Search strategy

**Table:**

**Population**	
1 (Child* adj3 (young or pre-school)).ti,ab. (72952) 2 child, preschool/ or infant/ (1228593) 3 (pregnan* or birth).ti,ab. (706117) 4 (neonat* or infant or perinatal or newborn).ti,ab. (517748) 5 exp Infant, Newborn/ (607223) 6 foetus.ti,ab. (7397) 7 Fetus/ (78154) 8 fetus.ti,ab. (64247) 9 (baby or babies).ti,ab. (69248) 10 exp Pregnancy/ or exp Pregnant Women/ (894010) 11 1 or 2 or 3 or 4 or 5 or 6 or 7 or 8 or 9 or 10 (2660727)	
**Intervention**	
12 ((clean* or modern) adj7 (energ* or fuel)).ti,ab. (2920) 13 (renewable* adj7 (energ* or fuel)).ti,ab. (4618) 14 (polic* adj7 (energ* or fuel)).ti,ab. (969) 15 (chang* adj7 (energy* or fuel)).ti,ab. (23059) 16 exp Renewable Energy/ or exp Biofuels/ (31360) 17 ((solar or wind or hydro*) adj5 (energ* or power*)).ti,ab. (19581) 18 (behavio$r adj9 (fuel or cook* or vent*)).ti,ab. (2091) 19 (transition adj7 (energ* or fuel)).ti,ab. (8426) 20 (electricit* adj7 energ*).ti,ab. (932) 21 ((hous* or home or domestic) adj7 (energ* or fuel)).ti,ab. (2213) 22 low polluting fuel*.ti,ab. (3) 23 (air adj7 ventilation).ti,ab. (3055) 24 (air pollution adj7 intervention).ti,ab. (73) 25 chimney.ti,ab. (1420) 26 “outdoor cook*”.ti,ab. (9) 27 12 or 13 or 14 or 15 or 16 or 17 or 18 or 19 or 20 or 21 or 22 or 23 or 24 or 25 or 26 (93856)	
**Household air pollution**	
28 ((household or indoor) adj3 air).ti,ab. (6933) 29 (HAP or IAP).ti,ab. (11561) 30 exp Air Pollution, Indoor/ (13569) 31 exp Particulate Matter/ (62709) 32 (“particulate matter” or “black carbon”).ti,ab. (19909) 33 exp Carbon Monoxide/ (17931) 34 “carbon monoxide”.ti,ab. (26660) 35 ((solid fuel or wood or charcoal or cook*) adj3 smok*).ti,ab. (1071) 36 (cookstove or stove).ti,ab. (1014) 37 Cooking/mt [Methods] (2210) 38 Household Articles/ (2254) 39 exp “Cooking and Eating Utensils”/ (1231) 40 26 or 27 or 28 or 29 or 30 or 31 or 32 or 33 or 34 or 35 or 36 or 37 or 38 or 39 (222736)	
**LMICs**	
41 (LMIC or “lower adj3 middle income” or “developing countr*”).ti,ab. (60345) 42 exp Developing Countries/ (74829) 43 exp Africa, Western/ or exp Africa, Northern/ or South Africa/ or exp Africa, Eastern/ or exp Africa, Central/ or exp “Africa South of the Sahara”/ or exp Africa/ or exp Africa, Southern/ (266418) 44 Africa.ti,ab. (109048) 45 exp South America/ (161665) 46 exp Asia, Central/ or exp Asia, Northern/ or exp Asia/ or exp Asia, Western/ or exp Asia, Southeastern/ (835914) 47 south America.ti,ab. (14583) 48 Latin America.ti,ab. (13762) 49 Asia.ti,ab. (59583) 50 (Afghanistan or Albania or Algeria or Angola or “Antigua and Barbuda” or Argentina or Armenia or Azerbaijan or Bangladesh or Belarus or Belize or Benin or Bhutan or Bolivia or “Bosnia and Herzegovina” or Botswana or Brazil or Burkina Faso or Burundi or Cabo Verde or Cambodia or Cameroon or Central African Republic or Chad or China or Colombia or Comoros or Democratic Republic of Congo or Congo or Cook Islands or Costa Rica or Cote d'Ivoire or Cuba or Djibouti or Dominica or Dominican Republic or Ecuador or Egypt or El Salvador or Equatorial Guinea or Eritrea or Ethiopia or Fiji or Gabon or Gambia or Georgia or Ghana or Grenada or Guatemala or Guinea or Guinea-Bissau or Guyana or Haiti or Honduras or India or Indonesia or Iran or Iraq or Jamaica or Jordan or Kazakhstan or Kenya or Kiribati or Democratic People's Republic of Korea or Kosovo or Kyrgyzstan or Lao People's Democratic Republic or Lebanon or Lesotho or Liberia or Libya or Former Yugoslav Republic of Macedonia or Madagascar or Malawi or Malaysia or Maldives or Mali or Marshall Islands or Mauritania or Mauritius or Mexico or Micronesia or Moldova or Mongolia or Montenegro or Montserrat or Morocco or Mozambique or Myanmar or Namibia or Nauru or Nepal or Nicaragua or Niger or Nigeria or Niue or Pakistan or Palau or Panama or Papua New Guinea or Paraguay or Peru or Philippines or Rwanda or Saint Helena or Samoa or “Sao Tome and Principe” or Senegal or Serbia or Sierra Leone or Solomon Islands or Somalia or South Africa or South Sudan or Sri Lanka or Saint Lucia or “Saint Vincent and the Grenadines” or Sudan or Suriname or Swaziland or Syrian Arab Republic or Tajikistan or Tanzania or Thailand or Timor-Leste or Togo or Tokelau or Tonga or Tunisia or Turkey or Turkmenistan or Tuvalu or Uganda or Ukraine or Uzbekistan or Vanuatu or Venezuela or Vietnam or “Wallis and Futuna” or “West Bank and Gaza Strip” or Yemen or Zambia or Zimbabwe).ti,ab. (1000461) 51 41 or 42 or 43 or 44 or 45 or 46 or 47 or 48 or 49 or 50 (1807589)	
**Grouped terms**	
52 11 and 27 (2227) 53 40 and 51 (25902) 54 11 and (27 or 53) (4306)	

## Abbreviations

ALRI: Acute lower respiratory infection
ARI: Acute respiratory infection
CENTRAL: The Cochrane Central Register of Controlled Trials
CO: Carbon monoxide
EPHPP: Effective Public Health Practice Project
GIM: Global Index Medicus
HAP: Household air pollution
ICS: Improved cookstove
ICTRP: International Clinical Trials Registry Platform
IUGR: Intrauterine growth retardation
LMICs: Low- and middle-income countries
LPG: Liquefied petroleum gas
RCT: Randomised control trial
PM: Particulate matter
PICOS: Population-Intervention-Comparator-Outcome-Study design
PRISMA: Preferred Reporting Items for Systematic Reviews and Meta-Analyses
PRISMA-P: Preferred Reporting Items for Systematic Reviews and Meta-Analyses Protocol
PROSPERO: International Prospective Register of Systematic Reviews
WHO: World Health Organization
